# Measuring Vulnerability and Resilience in the Time of Change Using

**DOI:** 10.23889/ijpds.v8i2.2185

**Published:** 2023-09-14

**Authors:** Ffion Lloyd-Williams, Stephen Drinkwater, Ffion Lloyd-Williams

**Affiliations:** 1 Welsh Government, Cardiff, United Kingdom; 2 Roehampton University, London, United Kingdom

## Objectives

Funded by ADR UK (ESRC) from 2020 to 2023, the project aims to anonymously link European Union Settlement Scheme (EUSS) data with other data already held within the SAIL Databank, based at Swansea University, and produce a research-ready dataset that can be used by researchers to obtain policy-relevant findings.

## Method

Using a range of de-identified data in the SAIL Databank, a control group of British citizens in Wales has been matched with EU citizens with similar characteristics using the Census 2011 as a spine to identify country of birth. The SQL coding programme is used to link Census data with several other datasets in the SAIL Databank relating to health, with a focus on mental health, education and employment. The R software package is used for statistical analysis to produce comparisons between the groups and performing significance tests of these comparisons (e.g. Mann Whitney).

## Results

Initial findings indicate small but statistically significant differences in school attendance between British pupils in Wales and pupils from EU14 and EU8 countries and similar differences in school attainment between British pupils in Wales and pupils from EU14 and EU8 countries. Further analysis has been conducted to explore differences between pupils from EU14 and EU8 countries. Census data are also linked to GP attendance data to explore differences in mental health related consultations and referrals for British citizens in Wales and citizens from EU countries and differences between citizens from EU14 and EU8 countries. Detailed findings from this linking of datasets and analysis will be presented.

## Conclusion

Linking data in this way helps to gain a better understanding of the experiences and outcomes of EU citizens in Wales, generating better evidence to help inform policies and services that address the needs of this population and offers a dataset of great interest to academics.

